# *Bacillus thuringiensis* subsp. *israelensis* and Its Dipteran-Specific Toxins

**DOI:** 10.3390/toxins6041222

**Published:** 2014-03-28

**Authors:** Eitan Ben-Dov

**Affiliations:** Department of Life Sciences, Achva Academic College, Mobile Post Office Shikmim 79800, Israel; E-Mail: etn@bgu.ac.il; Tel.: +972-8-6479037; Fax: +972-8-6472983

**Keywords:** biological control, mosquito-borne diseases, larvicidal crystal proteins

## Abstract

*Bacillus thuringiensis* subsp. *israelensis* (*Bti*) is the first *Bacillus thuringiensis* to be found and used as an effective biological control agent against larvae of many mosquito and black fly species around the world. Its larvicidal activity resides in four major (of 134, 128, 72 and 27 kDa) and at least two minor (of 78 and 29 kDa) polypeptides encoded respectively by *cry4Aa*, *cry4Ba*, *cry11Aa*, *cyt1Aa*, *cry10Aa* and *cyt2Ba*, all mapped on the 128 kb plasmid known as pBtoxis. These six δ-endotoxins form a complex parasporal crystalline body with remarkably high, specific and different toxicities to *Aedes*, *Culex* and *Anopheles* larvae. Cry toxins are composed of three domains (perforating domain I and receptor binding II and III) and create cation-selective channels, whereas Cyts are composed of one domain that acts as well as a detergent-like membrane perforator. Despite the low toxicities of Cyt1Aa and Cyt2Ba alone against exposed larvae, they are highly synergistic with the Cry toxins and hence their combinations prevent emergence of resistance in the targets. The lack of significant levels of resistance in field mosquito populations treated for decades with *Bti*-bioinsecticide suggests that this bacterium will be an effective biocontrol agent for years to come.

## 1. Introduction

Mosquitoes are an enormous public health menace in transmitting various tropical diseases and generally as a nuisance [1]. Many species of the genera *Anopheles*, *Aedes* and *Culex* are vectors of, e.g., malaria, yellow fever, dengue fever, hemorrhagic fever and lymphatic filariasis [2,3,4]. Despite the use of synthetic pesticides over the past 70 years, mosquito-borne diseases are still threatening half of the world's population. Malaria remains one of the leading causes of morbidity and mortality and kills about 660,000 people a year, mainy young children in Africa [5]. Chemical insecticides used in vector control programs harm the environment with adverse impacts on man and nature. Resistance to such insecticides among mosquito species that are vectors of malaria (*Anopheles gambiae*) and West Nile virus (*Culex pipiens*) emerged over 25 years ago in Africa, America and Europe and it is frequently due to loss of sensitivity of the insect's acetylcholinesterase to organophosphates and carbamates [6]. Alternative technologies such as biological control offer alternatives to deal with these problems and limitations [7].

## 2. The Bacterium: *Bacillus thuringiensis* subsp. *israelensis*

**Figure 1:**
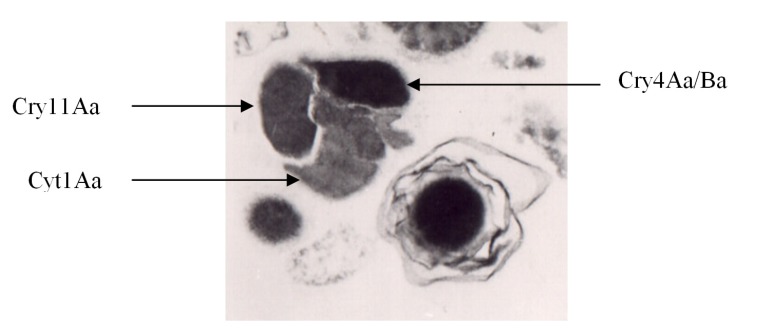
*B. thuringiensis* subsp. *israelensis*: crystal (left) and spore (right). Modified from Manasherob *et al.* [49].

*Bacillus thuringiensis* subsp. *israelensis* (*Bti*) is the first subspecies of *B. thuringiensis* (*Bt*) found to be toxic to dipteran larvae. This gram-positive spore-forming subspecies is the most powerful and environmental-friendly biological alternative component in integrated programs to control disease vectors [8,9]. *Bti* forms a crystalline parasporal body composed of protein protoxins (δ-endotoxins) (Figure 1) that are also used as a commercial bio-pesticide against larvae of noxious arthropod species of the suborder *Nematocera*, including mosquitoes, black flies and chironomid midges [7,9]. *Bti* is much more effective against many species of mosquito and black fly larvae than any previously known bio-control agent. Resistance to *Bti* extensively searched for in field populations of mosquitoes, has not been detected despite nearly 35 years of extensive field usage [10,11,12,13,14]. Several recent studies reported decreased susceptibilities in some field populations [15,16,17,18], but natural variation in such populations and different laboratory strains as well as technical variations inherent in bioassay tests need to be considered in interpreting bioassay results [19]. Thus, lethal concentration values that differ by 5-fold or less are not likely to reliably indicate resistance, and as a general guideline, differences of at least 10-fold are necessary for proof of resistance [15]. The lack of resistance to *Bti* is mainly attributed to different modes of action and synergistic interactions between the four major toxins, Cry4Aa, Cry4Ba and Cry11Aa and Cyt1Aa [20,21,22]. 

In addition to mosquitoes, black flies [23] and chironomid midges [24,25] the expanded host range of *Bti* includes the following species: *Tabanus triceps* (Diptera: Tabanidae) [26], Mexican fruit fly, *Anastrepha ludens* and Mediterranean fruit fly, *Ceratitis capitata* (Diptera: Tephritidae) [27,28], *Tipula paludosa* (Diptera: Nematocera) [29], fungus gnats, *Bradysia coprophila* and *Bradysia impatiens* (Diptera: Sciaridae) [30,31], nodule-damaging fly *Rivellia angulata* (Diptera: Platystomatidae) [32], pea aphid *Acyrthosiphon pisum* (Hemiptera: Aphidoidea) [33], potato aphid, *Macrosiphum euphorbiae* (Homoptera: Aphididae) [34], cotton boll weevil *Anthonomus grandis* (Coleoptera: Tenebrionidae) [35], leaf beetle, *Chrysomela scripta* (Coleoptera: Chrysomelidae) [36], fall armyworm, *Spodoptera frugiperda* (Lepidoptera: Noctuidae) [37], diamondback moth, *Plutella xylostella* (Lepidoptera: Plutellidae) [38], root-knot nematode, *Meloidogyne incognita* on barley [39] and trematode species, *Schistosoma mansoni* and *Trichobilharzia szidati* (Trematoda: Schistosomatidae) [40].

Originally isolated from a temporary pond with dying *Cx. pipiens* larvae [41], *Bti* seems able to reproduce and persist under natural conditions [42,43,44]. Spayed suspension of *Bti* (spores and crystals) settles within 24–48 h at the bottom of mosquito breeding sites. Ingested spores germinate and recycle in carcasses of *Bti*-killed mosquito larvae [45,46,47] and pupae [48], and the carcasses are toxic to mosquito larvae.

Other organisms coexisting in mosquito breeding sites may support *Bti* multiplication in nature, e.g., the ciliate protozoan *Tetrahymena pyriformis* [49]. Spores and δ-endotoxins are not destroyed in *T. pyriformis* during the digestion process; the spores germinate in excreted food vacuoles and complete a full growth and sporulation cycle in them [49,50]. In the absence of mosquito larvae, some recycling was observed in laboratory experiments with sediments and vegetation [42], in which case the persistence pattern of the δ**-**endotoxin components (Cry4 > Cry11 > Cyt) differs from that of the *Bti* parasporal body crystals [44].

## 3. δ-Endotoxins of *Bti*

The original isolate of *Bti* harbors eight circular plasmids ranging in size between 5 and 210 kb and a linear replicon of approximately 16 kb [51,52]. The larvicidal activity of *Bti* resides in at least four major crystal protoxins, of 134, 128, 72 and 27 kDa, encoded by *cry4Aa*, *cry4Ba*, *cry11Aa* and *cyt1Aa* respectively, all mapped on the 128 kb plasmid known as pBtoxis [53,54,55]. In addition, pBtoxis contains *cry10Aa*, *cyt2Ba* and *cyt1Ca*: Cry10Aa and Cyt2Ba accumulate in small amounts in the parasporal body and seem to contribute to the overall toxicity of *Bti* [56,57,58]. The large protoxins (Cry4Aa and Cry4Ba) have conserved C-terminal halves participating in spontaneous crystal formation via inter- and intra-molecular disulphide bonds [59,60], whereas the smaller (Cry11Aa and Cyt1Aa) do not possess this domain and hence require assistance in crystallization [61,62,63]. The *cry11Aa* is organized in an operon together with *p19* and *p20* [64,65]. The P20 accessory protein stabilizes both Cyt1Aa and Cry11Aa in recombinant *Escherichia coli* [61,62,63,66,67], *Pseudomonas putida* [63] and *Bt* [68,69] by interactions with the nascent polypeptides thus protecting these protoxins from proteolysis [61,62,63]. 

The Cry and Cyt toxins are membrane-perforating proteins although unrelated structurally and differ in their requirement of essential membrane components; the Cry’s bind to membrane receptors [70,71,72,73,74,75] whereas Cyt1Aa binds with high affinities to unsaturated phospholipids [76,77].

### 3.1. Major Toxins

Cry4Aa, encoded by a sequence of 3543 bp (1180 amino acids), is highly toxic to larvae of *Culex* and less to *Anopheles* and *Aedes* [19,21,70,78,79], and Cry4Ba, encoded by a sequence of 3408 bp (1136 amino acids), has high larvicidal activities against *Anopheles* and *Aedes* but very low against *Culex* [19,21,70,78,79]. Consistent with the differential specificities, the identity between the amino acid sequences of their N-termini toxic portions is only about 30% (55% similarity) [80,81]. Cry11Aa is encoded by a sequence of 1929 bp (643 amino acids) and displays high larvicidities against the larvae of *Aedes* and *Culex* but lower against *Anopheles* [19,21,82].

The larvicidal activity of Cyt1Aa, encoded by a sequence of 744 bp (248 amino acids), is low against all three mosquito genera [19,83,84]. It is cytolytic *in vitro* to cells of certain vertebrates and invertebrates [85] and highly mosquito species-specific* in vivo*, implying a specific mode of action [83,86]. The cytotoxicity seems to derive from an interaction between its hydrophobic segment and membrane phospholipids. The sequence of Cyt1Aa has no homology to Cry polypeptides [87] but the toxins plays a critical role in delaying selection for resistance to *Bti*’s Cry proteins [22,88,89,90].

### 3.2. Minor Toxins

Cry10Aa, encoded by a sequence of 2025 bp (675 amino acids), accumulates to minor amounts in *Bti* crystals [57,91] and differs markedly from Cry4Aa and Cry4Ba. The *cry10Aa* is arranged in an operon 48 bp upstream *orf2* [55]. Orf2 is highly homologous (over 65%) to sequences at the carboxylic end of Cry4Aa and Cry4Ba. It can be speculated that together, *cry10Aa* and *orf2* is a variant of the *cry4*-type genes. Toxicity of Cry10Aa is comparable to those of the other Cry4 toxins and is synergistic with Cyt1Aa against *Aedes aegypti* larvae [92] and with Cry4Ba against *C. pipiens* larvae [93].

Cyt2Ba, encoded by a sequence of 789 bp (263 amino acids), is found at very low concentrations in *Bti* crystals [56]. Proteolytically activated Cyt2Ba is hemolytic *in vitro* [94,95] and exhibits lower toxicities against larvae of *Culex, Aedes* and *Anopheles* than Cyt1Aa [94] but higher than Cyt1Ab from *Bt* subsp. *medellin* [96]. Cyt2Ba is synergistic with Cry4Aa, Cry4Ba or Cry11Aa [97,98] and with the *B sphaericus* binary toxin [96]; it may thus contribute to the overall toxicity of *Bti*.

Cyt1Ca is encoded by sequence of 1575 bp (525 amino acids) [98,99]. Its N-terminal half is 52% identical to Cyt1Aa, and at the C terminus it contains an extra domain, which appears to be a *β*-trefoil carbohydrate-binding motif, similar to the receptor binding domain of ricin-B lectin type found in several ricin-like toxins [99]. Transcripts of *cyt1Ca* were detected, but Cyt1Ca has not been found [100]; the reason may include instability of the transcript or the protein and failure in message translation. Neither mosquito larvicidal activity nor other biological function has been reported for Cyt1Ca [98,99]. The lack of activity of Cyt1Ca may be related to its inability to undergo a certain conformational change due to its lack of flexibility [101].

### 3.3. Activation, Three-Dimensional Structure and Mode of Action of Major Cry Toxins

Basic studies of the structures and modes of action of δ-endotoxins and their receptors are important for future development of biopesticides that will not be prone to insect resistance [74]. The level of toxicity depends on the capacity of the target species to activate the protoxin by cleaving it to the active toxic component(s) using specific proteases under the alkaline conditions prevailing in the larval midgut. The activated Cry4Aa and Cry4Ba are ~65 kDa toxins with three distinct-domains. The N-terminal domain I is a seven helix bundle responsible for pore formation, and the following two resemble carbohydrate binding proteins: a β-prism (domain II) and a plant lectin-like β-sandwich (domain III) [81,102,103,104,105]. Their three-dimensional structures are similar to the tertiary structures of other previously-solved Cry’s [106,107,108]. *In vitro* and *in vivo* processing yields two fragments, of 45 and 20 kDa for Cry4Aa and 45 and 18 kDa for Cry4Ba [79,109,110]. Processing of Cry11Aa yields similarly two fragments, of 38 and 30 kDa [79,82,111,112,113]. This mode of processing differs from those of the lepidopteran-specific toxins [114].

Subsequent steps involve toxin binding to receptors [72,73,74,75,104,105,114,115,116], oligomerization and membrane insertion leading to formation of gated, cation selective channels [117,118]. Lethality is due to collapse of the trans-membrane potential, with subsequent osmotic lysis of cells lining the midgut [119]. These three toxins bind *in vitro* to the apical brush border of midgut cells in the gastric caecae and posterior stomach of *An*. *gambiae* larvae [120] and to the midgut microvilli of *Ae. aegypti* [79]. The same cells in *Cx*. *pipiens* bind Cry4Aa specifically (both *in vitro* and *in vivo*) [75]. Each of the two Cry4Aa fragments of 20 (domain I) and 45 kDa (the α6 and α7 helices of domain I and domains II and III), produced by the intramolecular cleavage of the 65-kDa intermediate, are separately not toxic against larvae of *Cx*. *pipiens*, but together they display significant toxicity through association with each other to form an active complex of apparently 60 kDa [109,110].

Different mosquito larvicidal activity spectra of both activated Cry4Aa and Cry4Ba likely stem from the structural differences found within domain II and distinct sites binding to the host receptors [70,81,105]. Domain II consists of three anti-parallel β-sheets packed by a central hydrophobic core and three surface-exposed loops at the apex of the domain which are thought to be involved in receptor binding. Loops 2 and 3 of Cry4Aa are an important determinant of the specific toxicity against larvae of *Aedes*, *Anopheles* and *Culex* [105,121], whereas in Cry4Ba, loops 1 and 2 specify the toxicity for *Anopheles* and *Aedes* [70,105,122]. Aminopeptidase N and alkaline phosphatase, anchored to glycosyl-phosphatidyl-inositol (GPI) in the epithelial membrane of the *Ae. aegypti* larval midgut were identified as the receptors of Cry4Ba [123,124], and α-amylase was identified as such in the midgut brush border membrane vesicles of *Anopheles albimanus* [125]. Cry4Aa may contains multiple sub-sites spread out in domains II and III that cooperate for receptor binding and thus differ from other well-characterized Cry toxins of *Bt* in their receptor binding mechanism(s) [126].

Pre-pore trimeric structures of either Cry4Aa or Cry4Ba seem to form in aqueous solution and in lipid monolayer, which may facilitate insertion of their three *α*4-*α*5 hairpins into the membrane [104,127,128]. Proteolytically activated Cry4Ba *in vitro* can also form pre-pore oligomers that are proficient in perforation and formation of stable ion channels even without support of the receptors [129].

The three-dimensional structure of Cry11Aa has still not been solved but an *in silico* model was obtained based on that of Cry2Aa [115]. The pattern of the protoxin activation involves specific proteolytic removal of 27 N-terminal residues and intra-molecular cleavage into two fragments of about 30–33 and 34–36 kDa. Coexistence of the two fragments is essential for toxicity against larvae of *Cx*. *pipiens* and *Culex quinquefasciatus* [111,112,113]. Cry11Aa binds specifically to 148 kDa and 78 kDa proteins of brush border membrane vesicles of *An. stephensi* and *Tipula oleracea* respectively [72]. Its putative receptors were identified as GPI-aminopeptidase N, GPI-alkaline phosphatase, cadherin and α-amylase [71,73,125,130,131]. Cry11Aa-receptor interaction seems to involve at least three exposed regions of domain II (loop α-8, β-4 and loop3) [115]. Loop α-8 plays a significant role in the interaction of the toxin with its receptor and subsequent toxicity [115].

### 3.4. Activation, Three-Dimensional Structure and Mode of Action of Cyt1Aa and Cyt2Ba Toxins

The crystal structure of the proteolytically activated, monomeric forms of Cyt2Ba and Cyt1Aa were solved to 1.8 Å and 2.2 Å resolutions, respectively [101,132]. The toxins are composed of a single pore-forming domain of α/β architecture with a β-sheet surrounded by two α-helical layers representing a cytolysin fold. This structure is strikingly similar to those of the protoxin form of Cyt2Aa from *Bt* *kyushuensis* [133] and the fungal volvatoxin A2 [134], suggesting that the toxic monomer of these proteins has a similar mode of activity against cell membrane.

Based on its structure, toxicity of Cyt1Aa is correlated with ability to undergo conformational changes that must occur prior to membrane insertion and perforation [101,132]. The cytolysin fold allows the α-helical layers to swing away, exposing the β-sheet to insert into the membrane. The putative lipid binding pocket between the β-sheet and the helical layer of Cyt1Aa and the hemolytic activity of Cyt1Aa, which resembles that of the pore-forming agents α-toxin and saponin, support this mechanism [101].

Cyt1Aa do not bind specific receptors but have strong binding affinity to the unsaturated fatty acids that compose the membrane of midgut epithelial cells of dipterans [77,135]. *In vitro* processing of Cyt1Aa protoxin yields single active 22–25 kDa fragment [136,137] that is about three times more effective than the protoxin [138,139].

Cyt1Aa binds to the apical brush border of midgut cells, to the gastric caecae and to stomach cells of *An. gambiae* larvae; this may be related to the ability of the toxin to perforate cell membranes without participation of any specific receptor [120] by a mechanism that is still a subject of debate. A higher proportion of unsaturated phospholipids in diptera than in other insects may be the reason for a greater affinity of Cyt δ-endotoxins to dipteran cell membranes and activity *in vivo*. This implies a specific mode of action that is different to those of *Bti* Cry’s, but an insect-specific receptor may still be essential for the specificity of the Cyt toxins [133,140].

Two different models were proposed for the mode of action of Cyt toxins: pore-forming [141,142] and detergent-like [143]. According to the former, Cyt binds as a monomer which then undergoes conformational changes, its C-terminal half composed mainly of β-strands is inserted into the membrane and the N-terminal half comprising mainly α-helices is exposed on the outside of the membrane [101,144]. Oligomerization on the cell membrane forms β-barrel pores [133,144,145] that induce equilibration of ions and net influx of water, cell swelling, and eventual colloid-osmotic lysis [117,119,146]. Consistent with a detergent-like mechanism, Cyt1Aa is rather adsorbed onto the surface as aggregates thereby causing nonspecific defects in membrane lipid packing, through which intracellular molecules can leak by all-or-nothing mechanism [138,139,143]. Both models may coexist if one considers a differential activity under different doses (concentration × time) [147]: specific perforation occurs at low toxin concentration or short exposure, whereas membrane disruption occurs at high levels or long times.

## 4. Synergy between the Toxins and Resistance of Targets

### 4.1. Synergistic Interactions between Bti δ-Endotoxins

The high, specific mosquito larvicidal properties of *Bti* δ-endotoxins are attributed to complex interactions between six proteins, Cry4Aa, Cry4Ba, Cry10Aa, Cry11Aa, Cyt1Aa and Cyt2Ba, differing in toxicity levels and against different species of mosquitoes (Table 1) [19,20,21,22,92,93,96,148]. Toxicity of each of the four Cry’s is higher than of the Cyt’s, but the high activity of the whole crystal results in synergies among them [19,20,21,22,92,98]. The combinations of Cry4Aa and Cry4Ba, of Cry4Aa and Cry11Aa, or of the three Cry’s, are synergistic against larvae of *Culex*, *Aedes* and *Anopheles* [19,20,21,66,78,149], whereas Cry4Ba and Cry11Aa are synergistic against *Ae*. *aegypti* [20]. Two minor crystal toxins, Cry10Aa and Cyt2Ba contribute to the insecticidal activity of *Bti* by synergistic interactions: Cry10Aa with Cyt1Aa against *Ae. aegypti* [92] and with Cry4Ba against *Cx. pipiens* [93] and Cyt2Ba with Cry4Aa against *Ae. aegypti* [98]. Despite the low toxicities of Cyt1Aa and of Cyt2Aa of *Bt* *kyushuensis* against exposed larvae, they are highly synergistic with the *Bti* Cry toxins and their combinations [20,22,88,89,90,150,151,152,153,154,155,156,157,158,159]. Each functions as a receptor for Cry4Ba, which binds through its domain II loops, explaining the synergy mechanism [155,159].

The suggestion that Cyt1Aa synergizes Cry11Aa by facilitating the latter’s interaction with the target cell or translocation of the corresponding toxic fragment [157] was later confirmed [154,158]: the interaction is based on their binding as follows. Cyt1Aa, which functions as a membrane-bound receptor, inserts its β-sheet into the membrane after conformational changes, two of its components (loop β6-αE and part of β7) bind with high affinity to Cry11Aa, which subsequently is inserted into the larval epithelial membranes [114,154,158]; residues K198 on β7, and E204 on α6 and K225 on β8 are involved in this process. Inconsistent with this model, these three residues seem to be inserted into the cell’s membrane [101], and an alternative mechanism suggests that Cry11Aa binds to Cyt1Aa using these exposed, charged residues prior to its membrane insertion. This mechanism was recently confirmed [160]: the synergy is retained in mutants of Cyt1Aa helix α-C that were affected in oligomerization, membrane insertion, hemolytic and insecticidal activities. Binding between Cyt1Aa and Cry11Aa may occur in solution or in the membrane plane, promoting oligomerization of Cry11Aa and thus synergizing its toxicity.

**Table 1:** *B. thuringiensis* subsp. *israelensis* crystal δ-endotoxins.

Toxin	Activated form (kDa)	Toxicity	Synergistic with
Cry4Aa (134 kDa)	20 and 45	Cx > An ≥ Ae	Cry4Ba, Cry11Aa, Cyt1Aa, Cyt2Ba
Cry11Aa (72 kDa)	18 and 45	An ≥ Ae > Cx	Cry4Aa, Cry11Aa, Cry10Aa, Cyt1Aa, Cyt2Aa
Cry11Aa (72 kDa)	30–33 and 34–38	Ae ≥ Cx > An	Cry4Aa, Cry4Ba, Cyt1Aa, Cyt2Ba
Cyt1Aa (27 kDa)	22–25	Cx ≥ Ae > An	Cry4Aa, Cry4Ba, Cry11Aa, Cry10Aa
Cry10Aa (78 kDa)	58–68	Ae > Cx	Cyt1Aa, Cry4Ba
Cyt2Ba (29 kDa)	22.5	Cx ≥ Ae > An	Cry4Aa, Cry4Ba, Cry11Aa
Cyt1Ca (57 kDa)	ND	ND	ND

### 4.2. Resistance of Targets to Bti δ-Endotoxins

Field and laboratory resistance of *Cx.* *quinquefasciatus* and *Cx.* *pipiens* to *Bti* have been found, regardless of their origin or the level of selection pressure applied [15], but only insignificant levels of resistance were attained against *Ae. aegypti*. In both examples, resistance was unstable in the absence of larval selection pressure and declined by 50% over three generations. Under laboratory selection pressure against individual Cry4Aa, Cry4Ba and Cry11Aa or in their combinations, larvae of *Cx.* *quinquefasciatus* evolve variable levels of resistance and cross-resistance, but only negligible resistance emerged when selected against all four major toxins, three Cry’s and Cyt1Aa [88,89,90]. Moreover, in the presence of moderate Cyt1Aa concentrations, the strains resistant to these Cry toxins (without Cyt1Aa) retained their original wild type sensitivity levels even to this highly effective combination [15]. Thus, increasing the number of Cry toxins delayed the evolution of resistance, but including Cyt1Aa in the combination used for selection was essential to the process [15,22,88,89]. The synergy between Cyt1Aa and Cry’s is significantly high against resistant larvae [22,90] due to the unique feature of Cyt1Aa that serves as an additional receptor for *Bti* Cry’s. Genetically, *Cx.* *quinquefasciatus* evolve multiple-loci resistance to the *Bti* Cry toxins, but progeny of reciprocal crosses to a sensitive strain exhibited autosomal inheritance with intermediate levels of resistance [161]. The interactions in the diverse mixture of *Bti* δ-endotoxins, particularly with Cyt1Aa, allow a long term use of *Bti* as a biological control means against mosquitoes and black flies.

## 5. Antibacterial and Anticancer Activities of *Bti* δ-Endotoxins

Expression of *cyt1Aa* alone in recombinant acrystalliferous *Bt* *kurstaki* and in *E. coli* causes loss of colony-forming ability [68,162]; the latter cells arrest growth and DNA replication leading to strong nucleoid compaction and partial lysis [163,164]. These findings support the suggestion that, in addition to its membrane perforating activity, Cyt1Aa specifically disrupts nucleoid associations with the cytoplasmic membrane. Simultaneous, high-affinity interactions of Cyt1Aa with zwitterionic phospholipids as well as with DNA may enhance detachment of DNA from the membrane and hence affect nucleoid compaction [163]. Co-expression with *p20* (encoding a putative chaperonin) preserves cell viability [68,165]. Antibacterial activity of expressed N terminus-truncated Cyt1Ca in *E. coli* causes instant arrest in biomass growth and decreased viability [99].

Cyt1Aa, Cry4Ba and Cry11Aa, as well as two proteins (of 36 and 34 kDa) isolated from *Bti*, are antibacterial also exogenously, against *E. coli* and Gram-positive species (*Micrococcus luteus, Streptomyces chrysomallus* and *Staphyloccocus aureus*) [137,166,167]. Cyt1Aa is bactericidal for *E. coli*, whereas it is bacteriostatic for *S. aureus* as reflected in morphological changes and ion balance alteration [137]. Cyt1Aa may bind to the outer membrane of Gram-negative cells and easily penetrate the cytoplasmic membrane, whereas in Gram positive cells, it must cross the massive peptidoglycan layer before reaching the cytoplasmic membrane. Furthermore, Cyt1Aa can contribute to the antibacterial activity of some antibiotics through partial disruption of the outer membrane, enabling better penetration of the antibiotic [137].

Cry toxins from other *Bt* subspecies (*kurstaki*, *galleriae*, *tenebrionis*) are toxic to the anaerobic Gram-positive bacteria *Clostridium butyricum* and *Clostridium acetobutylicum* and the archaea *Methanosarcina barkeri* [168].

Cyt1Aa and Cyt2Ba display anti-cancer activities as well; conjugation of activated Cyt1Aa to a peptide carrier molecule is toxic against murine hybridoma cells [169] whereas activated Cyt2Ba exhibits some cytotoxicity to human breast cancer cells (MCF-7) [170]. Cyt1Aa may be useful in other medical applications: specific toxicity against cells bearing a high number of insulin receptors is enhanced by linking it to insulin [171].

## 6. Limitations of *Bti* and Recombinant Bacteria

Applying *Bti* for mosquito control is limited by short residual activity of current preparations under field conditions [9] due to: (i) sinking to the bottom of the water body; (ii) adsorption onto silt particles and organic matter; (iii) consumption by other organisms to which it is nontoxic; and (iv) inactivation by sunlight. In order to overcome these shortcomings, the δ-endotoxin genes have already been expressed individually or in combinations in various Gram-positive and -negative species [7,9]. Best results were achieved by expressing the genes encoding binary toxin of *B*. *sphaericus* in *Bti* [172]. The recombinant bacteria were highly potent against fourth instar larvae of *Cx. quinquefasciatus* and *Cx. tarsalis*, even to lines selected for resistance to the binary toxin. Higher toxicity against fourth-instar *Cx. quinquefasciatus* was achieved in recombinant acrystalliferous *Bti* strain that produces the combination of *B. sphaericus* binary toxin together with Cyt1Aa of *Bti* and Cry11Ba from *Bt* subsp. *jegathesan* [173].

Several attempts have been made during the last two decades to produce transgenic mosquito larvicidal cyanobacteria [9,174]. Most promising results were obtained when *cry4Aa* and *cry11Aa* alone or with *cyt1Aa* were expressed from the dual constitutive and efficient promoters *P_psbA_* and *P_A1_* in the filamentous, nitrogen-fixing cyanobacterium *Anabaena* PCC 7120 [174,175,176,177,178,179]. LC_50_ values of these clones against third and fourth instar *Ae. aegypti* larvae were 8.3 × 10^4^ and 3.5 × 10^4^ cells mL^−1^ respectively, the lowest reported values for engineered cyanobacterial cells with *Bti* toxin genes. Toxicity of the *Anabaena* clone expressing constitutively *cry4Aa* and *cry11Aa* with *p20* is retained following irradiation by high doses of UV-B, doses that partially inactivate *Bti* toxicity [177]. This latter recombinant strain exhibited decent toxicity against larvae of *An. merus*, *An. arabiensis* and *An*. *gambiae*, but very weak activity against *An*. *funestus* [178]. Optimizing growth conditions in a photobioreactor was described for this cyanobacterial clone [179].

## 7. Concluding Remark

*Bti* is environmentally friendly and a safe alternative means to control mosquitoes and blackflies. Emergence of resistant variants has not been found despite three decades of extensive use, likely due to the complex and diverse δ-endotoxins composition of its crystal. To overcome or prevent theoretical, future emergence of resistance, recombinant microorganisms can be engineered to co-express toxins with different modes of action or chimeric toxins with improved efficacy [180]. Enhancing *Bti*’s mosquito larvicidal activity can be achieved by totally different mechanisms that wane larval survival, e.g., chitinase (damaging the peritrophic membrane) [181] and Trypsin Modulating Oostatic Factor (causing larval starvation) [182,183].
